# The Role of Aging and Senescence in Immune Checkpoint Inhibitor Response and Toxicity

**DOI:** 10.3390/ijms25137013

**Published:** 2024-06-27

**Authors:** Sidharth S. Jain, Giselle Burton Sojo, Harry Sun, Benjamin N. Friedland, Megan E. McNamara, Marcel O. Schmidt, Anton Wellstein

**Affiliations:** Georgetown Lombardi Comprehensive Cancer Center, Georgetown University, Washington, DC 20007, USA; ssj36@georgetown.edu (S.S.J.);

**Keywords:** immune checkpoint inhibitor, aging, cellular senescence, immune-related adverse events

## Abstract

Cellular senescence accumulates with age and has been shown to impact numerous physiological and pathological processes, including immune function. The role of cellular senescence in cancer is multifaceted, but the impact on immune checkpoint inhibitor response and toxicity has not been fully evaluated. In this review, we evaluate the impact of cellular senescence in various biological compartments, including the tumor, the tumor microenvironment, and the immune system, on immune checkpoint inhibitor efficacy and toxicity. We provide an overview of the impact of cellular senescence in normal and pathological contexts and examine recent studies that have connected aging and cellular senescence to immune checkpoint inhibitor treatment in both the pre-clinical and clinical contexts. Overall, senescence plays a multi-faceted, context-specific role and has been shown to modulate immune-related adverse event incidence as well as immune checkpoint inhibitor response.

## 1. Introduction

Nearly 20 million diagnoses of cancer are made each year around the world, and 75% of these diagnoses are in individuals aged 55+ [1], suggesting that cancer is largely a disease of aging. Younger patients tend to present with hematological malignancies or other cancers linked to genetic alterations or early exposure to carcinogenic agents [2]. Older patients, on the other hand, are more likely to present with advanced, unresectable cancers [3,4], likely caused by an accumulation of cellular damage with age. Various causes of cellular damage lead to elevated cancer incidence, including but not limited to oxidative stress, metabolic dysfunction, chronic infection or inflammation, exposure to reactive oxygen species (ROS), and exposure to carcinogens [5].

The advent of immune checkpoint inhibitor (ICI) therapy has transformed clinical management of unresectable and metastatic solid and hematological malignancies [6,7]. As of June 2024, there are over 2000 clinical trials actively investigating the use of ICI for a variety of disease contexts [8]. We have previously reviewed existing ICI treatments currently in use in the clinic [9]. Although these treatments are extensively used in the treatment of various cancers, the predictive biomarkers for both response and toxicity to ICI remain scarce [10,11,12]. Widely used biomarkers for response, including PD-L1 expression and tumor mutational burden, achieve an area under the receiver operatic characteristic curve (AUC-ROC) between 0.51 and 0.74 [11,13], suggesting that key aspects of the biology underlying response to ICI remain enigmatic. Many patients on ICI treatment also experience immune-related adverse events (irAEs), where the treatment is believed to incite autoimmune-mediated damages of tissues. Many factors have been shown to influence the incidence of toxicities of ICI treatment, but the exact mechanisms of irAEs remain poorly understood [14,15,16].

Aging and cellular senescence have been shown to play a critical role in modulating the response to anti-cancer therapies in multiple contexts [17,18,19]. The extent of impact that aging and cellular senescence (hereafter referred to as senescence) have on ICI response and toxicity remains poorly understood and is an evolving area of research. Senescence can occur in tumor cells, in tumor-adjacent stromal cells, in normal tissue, and in the immune system, affecting multiple factors that govern ICI response.

In this review, we seek to explore the current understanding of the effect of senescence on immune checkpoint inhibitor response and toxicity. We provide an overview of senescence, review preclinical evidence of the potential mechanisms by which senescence and aging impact ICI response and toxicity, and highlight clinical findings that assess the relationship between aging, senescence, and ICI treatment.

## 2. Overview of Aging and Senescence

### 2.1. Aging Is a Result of Senescence

**Figure 1:**
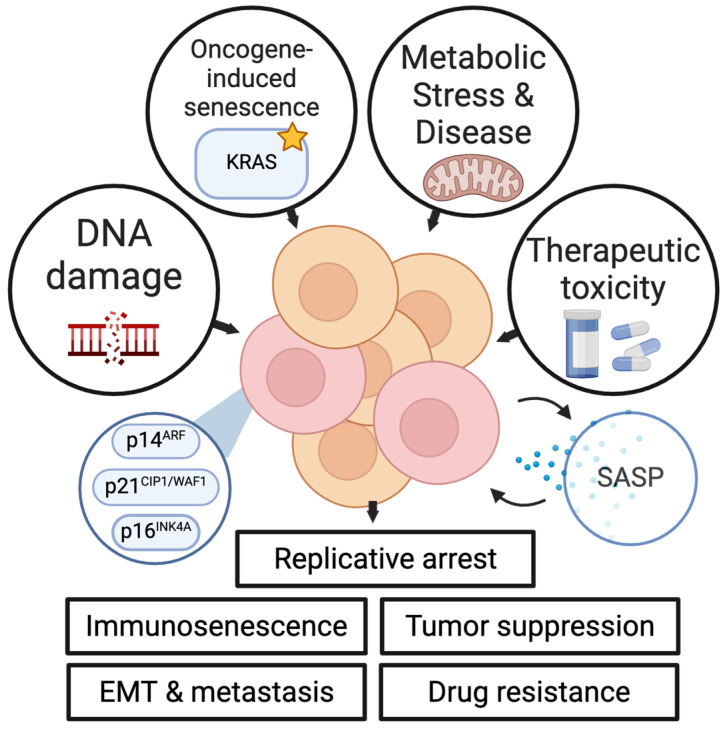
An overview of senescence causes and effects. Senescence can be induced by several factors, including accumulative DNA damage [32], oncogenic signaling (e.g., mutant KRAS, indicated by the star) [41], metabolic stress and diseases such as diabetes [40], and therapeutic toxicities from cytotoxic chemotherapy [18]. Senescence is driven at a transcriptional level by several key cell cycle proteins, including p16^INK4A^, p14^ARF^, and p21^CIP1/WAF1^ [30,31]. Senescence induction results in the production of the senescence-associated secretory phenotype (SASP), a collection of chemokines and cytokines expressed in senescent cells including IL-6, IL-8, IL-15, CXCL1, CCL3, and others [33]. Senescence can result in tumor suppression in pre-malignant cells [42] but can also result in treatment resistance [18,43], enhanced metastatic potential [20,44], and suppressed anti-tumor immune function through immunosenescence [45].

Aging has been implicated in multiple aspects of cancer initiation [20], progression [21], metastasis [22], and treatment [23]. Cellular senescence, a key biological driver of the phenotypes associated with aging, was first described by Weismann in 1881 when evaluating the principles that govern the duration of life and was subsequently re-discovered by Hayflick and Moorhead in the early 1960s [24]. It is defined as a transition to irreversible growth arrest of a cell [25]. Unlike quiescence, which is a reversible transition to the G0 phase of the cell cycle, senescence is believed to be irreversible, although there is some evidence that cells can transition out of senescence [26]. Senescence was initially characterized by the expression of hallmark senescence genes, namely p16^INK4A^ and p14^ARF^ (encoded by CDKN2A) and p21^CIP1/WAF1^ (encoded by CDKN1A). These hallmark genes are involved in cell cycle regulation and arrest [27,28,29,30] and can be induced by different mechanisms that include oncogene signaling, DNA damage, oxidative stress, and others (Figure 1). The inhibitory activity of p16^INK4A^ and p14^ARF^ leads to elevated p53 (TP53) activity and decreased phosphorylation of Rb (RB1), which results in cell cycle arrest [31,32] and subsequent senescence. Senescence is also characterized by the senescence-associated secretory phenotype (SASP), which involves the production of several secreted cytokines and chemokines that are produced by senescent cells [33,34,35]. Expression of SASP-related genes is at least in part driven by nuclear factor κB (NFKB1) signaling. In their study, Chien et al. showed that inhibition of NF-κB signaling in vitro in senescent cells decreased the mRNA expression of IL1A, IL6, CXCL1, and ICAM1, known components of the SASP [36]. At the chromatin level, the formation of senescence-associated heterochromatin foci has also been used as a key marker of senescence. These heterochromatin foci are mediated by the recruitment of Rb to E2F promoters [37]. Senescence-associated β-galactosidase [38], a lysosomal β-galactosidase, is a widely used indicator of cellular senescence of cultured cells or senescent cells in tissues. Though some studies have called into question the specificity of this indicator [39], other work has shown that selective elimination of cells expressing senescence-associated β-galactosidase results in the reversal of senescence-associated phenotypes [40].

### 2.2. Causes and Consequences of Senescence

Senescence can be brought on by insult to the cell, including DNA damage [34], oncogene activation [41,46], metabolic stress [47,48,49,50], reactive oxygen species and oxidative stress [47,48,51], and replication stress [52]. Loss of homeostasis in the context of these cellular stresses leads to cell cycle arrest, driven by p16^INK4A^ and p21^CIP1/WAF1^, and serves both protective and maladaptive roles in physiological function [53,54,55]. In normal development, p21-driven senescence plays a critical role in tissue remodeling during embryogenesis [56]. Controlled senescence is also required for proper wound healing [57]. In a study of wound healing, the secretion of PDGF-AA (homodimeric isoform encoded by PDGF), a component of the SASP, was required for wound healing in a preclinical mouse model of senescence [58], and ablation of senescent cells inhibited the wound-healing process. Senescence has also been shown to induce angiogenesis through the actions of SASP [26]. Our previous work also showed that angiotensin II was sufficient to induce senescence in kidney endothelial cells, and that selective clearance of senescent cells reverses downstream effects of senescence and prevents senescence-mediated immune infiltration [59]. Senescent cells characterized by p16^INK4A^ expression accumulate during physiologic aging. In addition to physiologic changes, multiple age-related pathologies (or “senopathies”) have been linked to the increased abundance of senescent cells [60], including osteoarthritis [61], pulmonary fibrosis [62], and atherosclerosis [63], to name a few.

**Figure 2:**
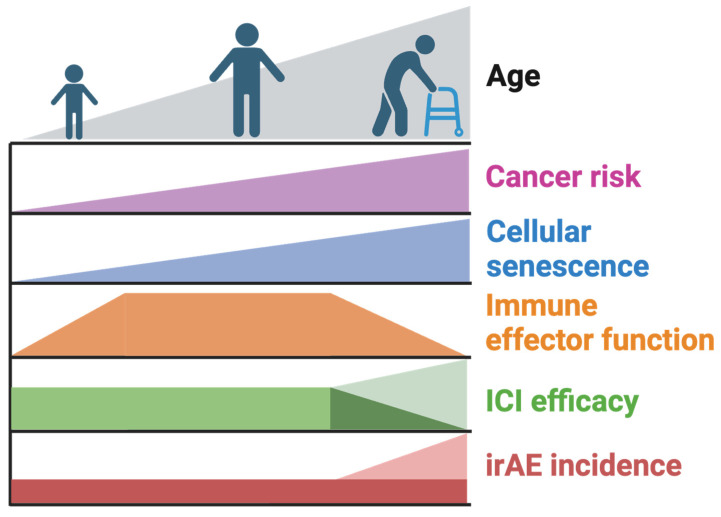
Summary of the age-associated trends relevant to immune checkpoint inhibitor (ICI) response and toxicity. With age, senescent cell burden and cancer risk increase colinearly. Immune effector function peaks during young adulthood and diminishes in elderly individuals. ICI efficacy has been demonstrated to both increase [76] and decrease [43] with aging and senescence, but in different contexts. The incidence of immune-related adverse events (irAEs), resulting from immune-mediated damage to normal tissues secondary to ICI therapy, is either similar [78,79,80] or elevated [81,82] in elderly patients compared to younger patients treated with immune checkpoint inhibitor therapy.

In the context of cancer, senescence plays a vital role in halting the proliferation of pre-malignant cells. Senescence is induced by oncogenic signaling—for example, BRAF V600 mutations are commonly found in non-malignant melanocytic nevi, which are induced to senesce through the activation of oncogenic BRAF signaling [64]. One of the key mediators of cellular senescence, p16^INK4A^, signals through the tumor suppressor Rb1 (RB1) to halt cell proliferation in cells undergoing potentially tumorigenic stress [42]. Indeed, the evolutionary pressures behind senescence include tumor suppressive function [65]. Though senescence is usually beneficial in the prevention of tumorigenesis, some effects of senescence may exacerbate tumorigenesis and enable tumor cell functions that promote malignancy. Trends of cellular senescence with age and related aspects of cancer onset and treatment response and toxicity are outlined in Figure 2. These aspects of cellular senescence are further elaborated below.

### 2.3. Therapeutic Modulators of Senescence

In their seminal work, Baker et al. demonstrated in a murine model that age-related phenotypes can be reversed through the clearance of p16-driven senescent cells [66]. This finding spurred a flurry of investigation into drugs that would specifically target and destroy senescent cells but leave normal proliferating or reversibly growth-arrested cells intact. Multiple drug repurposing studies proposed candidate compounds including dasatinib and quercetin [67], fisetin and curcumin [68], anti-apoptotic BCL2/BCL-xL inhibitors such as navitoclax [69,70], and others. These treatments and the history of their development have been extensively reviewed [71]. The results of several clinical trials evaluating senolytic therapies in patients with existing age-related pathologies have shown promising albeit modest results [72,73,74,75]. The potential impact of senolytic or senomodulatory therapy on the safety and efficacy of ICI treatment has not yet been evaluated, but encouraging preclinical evidence [76,77] suggests that this interaction may be worth investigating in a clinical trial.

## 3. Senescence and the Immune System

### 3.1. Immune Checkpoint Protein Expression in Different Cell Types

**Figure 3:**
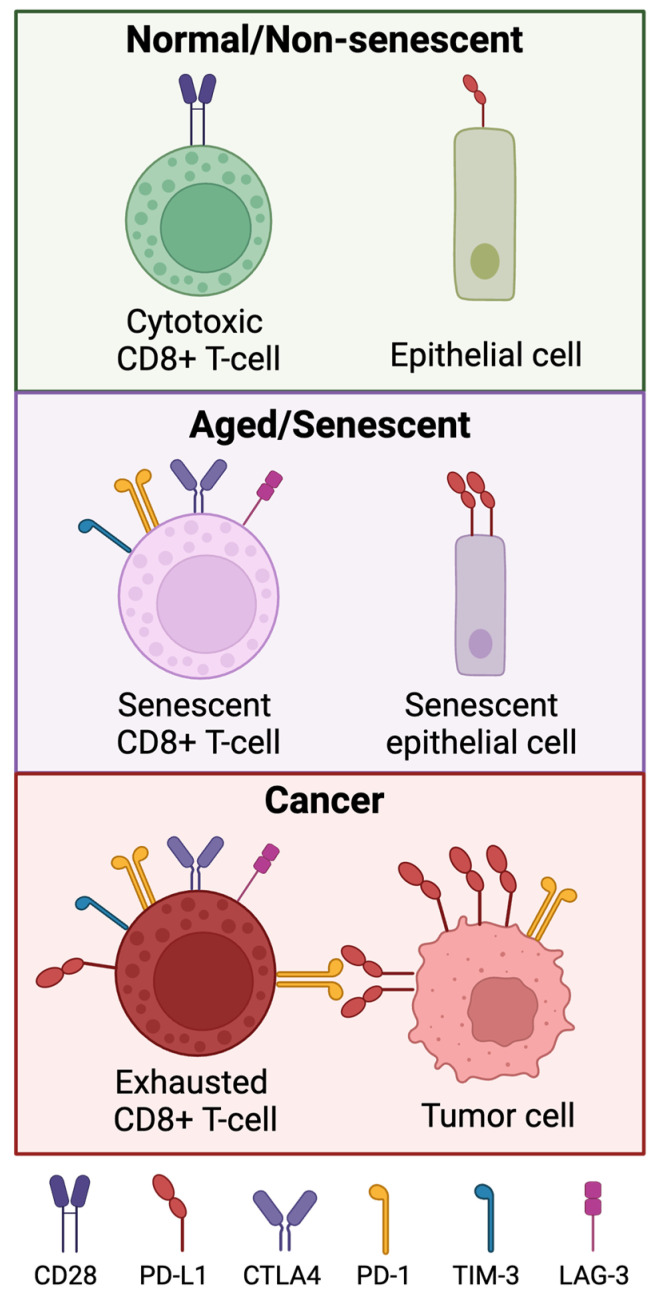
Expression of immune checkpoint proteins in normal, aged/senescent cancer cells. Senescent and tumor-associated T cells have loss of CD27 and CD28. Senescent immune cells express higher levels of PD-1, TIM-3, LAG-3, and CTLA4. Cancer-associated T cells also express these markers, and the interaction between PD-1 and PD-L1 contributes to the exhausted phenotype in tumor-associated T cells. Normal epithelial cells express very low levels of PD-L1, but senescent epithelial cells express PD-L1 at a notably higher level.

The immune checkpoint protein PD-1 (programmed death receptor 1) is present on exhausted T cells. Binding of PD-1 to its ligands, PD-L1 or PD-L2, suppresses T cell activity and proliferation [83]. PD-L1 is expressed on tumor cells as a mechanism of immune evasion [84]. Therapeutic disruption of the PD-1/PD-L1 interaction promotes PD-1+ T cell-mediated killing of PD-L1+ tumor cells. However, PD-1 is also expressed in other immune and non-immune cell types (Figure 3). Natural killer (NK) cells also express PD-1, and this expression appears to be age-dependent [85]. PD-1 expression on tumor cells has been highlighted as a mechanism of immunotherapy resistance [86]. PD-L1, similarly, is not only expressed on tumor cells. Immune cells in the tumor microenvironment have been shown to express PD-L1, including CD8+ T cells. The PD-L1+CD8+ T cell population plays a pro-tumorigenic role by suppressing effector T cells, but it also promotes self-tolerance [86], which may also partially explain the onset of irAEs in anti-PD1 therapy. At a basal level, other normal cell types, including microvascular endothelial cells and renal tubule epithelial cells, also express low levels of PD-L1 [86]. Aging and senescent cells have been observed to express PD-L1 more than younger cells of the same type, leading some to suggest the use of anti-PD1 or anti-PD-L1 ICI treatment as a potential senolytic agent [87,88].

Targeting the cytotoxic T-lymphocyte-associated protein 4 (CTLA4) immune checkpoint receptor has also proven to be an effective strategy in reversing T cell exhaustion. Normally, the interactions between CTLA4 and its binding partner B7 lead to stimulation and activation of lymphocytes [89]. Inactive T cells express higher levels of CTLA4, and abrogation of the interaction between CTLA4 and B7 resulted in reactivation in T cells [90,91]. However, like PD-1, CTLA4 is not exclusively expressed in exhausted T cells. In normal healthy pituitary cells, CTLA4 is expressed widely, leading to hypophysitis as a common immune-related adverse event in anti-CTLA4 treatment [92]. Aging also leads to an increase in the expression of CTLA4 on the surface of T cells [93], suggesting that T cell exhaustion and senescence-associated processes may be intertwined.

### 3.2. Immune-Related Adverse Events May Occur Systemically

Treatment with immune checkpoint inhibitor therapy can cause immune-related adverse events in nearly half of all patients [94]. These irAEs can affect nearly any tissue in the body, most commonly in skin, endocrine, and GI organs [95], resulting in symptoms including pruritis, hypophysitis, hypothyroiditis, nausea, vomiting, diarrhea, colitis, and many others. The most severe irAEs can also be life-threatening and include myocarditis [96]. Though the exact mechanisms of irAEs have not been identified, several observations suggest that irAEs occur due to a loss of self-tolerance and increased recognition of self-antigen, immune overstimulation resulting in increased systemic inflammation, expression of checkpoint proteins in non-tumor tissue, or other yet uncharacterized mechanisms. These and other hypotheses have been comprehensively reviewed elsewhere [14,78,96,97,98].

## 4. Senescence and the Immune System

### 4.1. Immune-Mediated Surveillance and Clearance of Senescence

Surveillance and clearance of senescent cells is a normal physiological function of the immune system. Proteins comprising the senescence-associated secretory phenotype act as paracrine and chemotactic signals, attracting circulating immune cells through the vascular endothelium into the tissue stroma [99]. Cytotoxic CD8+ T cells and NK cells have been shown to play a critical role in the clearance of senescent cells [100]. Immune-mediated surveillance of senescent cells is an important component of anti-tumor immunity [101]. Eggert et al. showed that oncogene-induced senescence in hepatocytes prevents hepatocellular tumorigenesis and stimulates the production of CCL2, a component of the SASP [45]. CCL2 stimulates immature circulating myeloid cells to differentiate into infiltrating macrophages that can subsequently clear senescent cells, and ablation of CCL2 led to hepatocellular carcinoma outgrowth.

### 4.2. Cellular Senescence Is a Component of Immunosenescence

Senescence can occur throughout the body, but of particular interest in the context of ICI therapy is the role of senescence in immune function and activation. As with other somatic cells, immune cells also undergo cellular senescence and other functional changes with age, resulting in an overall age-associated deterioration in immune function, termed immunosenescence [102]. Age-associated immune cell senescence has been noted in multiple immune compartments, with evidence that senescent CD8+ T cells were more abundant in older individuals, as well as in younger patients infected with human immunodeficiency virus [102]. Senescence in immune cells has been shown to be driven by p16^INK4A^, as in other somatic cells, which suggests that p16-driven clearance of senescent cells may also be effective [103].

Another key aspect of immunosenescence that is not encompassed by cellular senescence is the potential for immune cell anergy and exhaustion. Immune exhaustion anergy results from reduced co-stimulatory signals. The loss of costimulatory protein CD28 in T cells may in part explain age-associated reductions in T cell activity that define immunosenescence [104]. Immune cell exhaustion, on the other hand, is usually defined by an increased expression of immune checkpoint proteins on the surface of immune cells. These include PD-1 (PDCD1), CTLA4, TIM-3 (HAVCR2), LAG3, and TIGIT [105]. Expression of CD57 was initially believed to more specifically mark senescent T cells [106], but recent studies have demonstrated that the CD57+ T cell population is in fact capable of replicating in vivo [107] and in fact may play an important role in the response to anti-PD1 therapy [108].

Interestingly, many markers of T cell exhaustion have also been associated with aging. In a murine model, CD8+ T cells tended to express higher levels of PD1 and LAG3 in mice of advanced age compared to their younger counterparts, and these aged T cells transcriptionally resembled exhausted T cells [106]. An observational study in humans showed that CTLA4 was more expressed in CD4+ T cells from older people, and that expression of CTLA4 was also negatively correlated with costimulatory CD28 expression [93]. Another study showed that TIM-3 expression was observed in CD8+ cells in aged mice, and that these cells produce the anti-inflammatory cytokine IL-10 [107]. The mechanisms that link immune exhaustion, anergy, and senescence have yet to be completely elucidated, but taken together, the observations in these studies suggest that although immune cell senescence, anergy, and exhaustion are discrete processes, they all are associated with aging, possibly explaining age-associated immunosenescence.

Cellular senescence in non-immune cell types also plays a strong role in immune function and immunosenescence. In physiological aging, the SASP from stromal senescent cells has been shown to signal to the vascular endothelium to recruit CD4+ and CD8+ T cells expressing STAT1 [100]. The SASP acts on these cells to induce clearance of senescent cells, which constitutes a core physiological function of T lymphocytes [108]. Senescent cells have been shown to upregulate the expression of MICA and ULBP2, which bind to the NK cell receptor NKG2D [109]. Evasion mechanisms of senescent cells include upregulation of HLA-E expression by senescent cells, which were shown to dampen the ability of NK cells and CD8+ T cells to perform degranulation and infiltration functions critical for senescent cells clearance [110]. However, senescence in the tumor microenvironment may play an immunosuppressive role. Montes et al. showed that patient-derived T cells exhibit senescence-like properties after co-culture with a tumor cell line, including increased p16, p21, and p53 expression, shortened telomere lengths, and loss of CD27 and CD28 [111]. These tumor-induced senescent T cells slowed proliferation of other non-senescent T cells and produced regulatory cytokines including IL-6 and interferon gamma [111]. To summarize, immunosenescence can be impacted both by direct cellular senescence in the immune cell population and by senescence in non-immune cells capable of modulating immune function.

### 4.3. Inflammaging Is a Cause and Consequence of Immunosenescence

The onset of “inflammaging”, or chronic inflammation associated with age, is strongly associated with immunosenescence. Senescence results in the production of cytokines associated with chronic inflammation, termed the senescence-associated secretory phenotype (SASP) [33,34]. These cytokines have been shown to stimulate innate immune cells, including NK cells and macrophages, which are the primary effector cells of senescent cell clearance [108,109,112]. However, persistent chronic inflammation signaling, particularly in the innate immune compartment, is linked to age-related dysregulated immune function [113,114]. A decreased lymphoid and increased myeloid immune population is characteristic [114,115,116] of age-related immune changes and inflammaging.

Though the overall population and effector function of the lymphocyte population decreases with inflammaging, specific changes in T cell functions have also been demonstrated to contribute to age-related chronic inflammation. For example, CD4+ FOXP3+ regulatory T cells in aged C57/Bl6 mice were unable to negatively regulate IL-17-producing T cells, which are key drivers of chronic inflammation [117]. In both mice and humans, diminished CD8+ T cell function has been linked to increased abundance of semi-differentiated T “virtual memory” cells, which exhibit a strong senescence phenotype [118]. Exactly how these changes in lymphocyte function and abundance may impact ICI efficacy or toxicity has not been well studied, but conflicting clinical evidence suggests that age-related changes in these cell populations may or may not impact response to ICI.

## 5. Clinical Evidence of Aging Impacting Immune Checkpoint Inhibitor Safety and Efficacy

### 5.1. CIinical Observations Regarding the Impact of Aging on Efficacy

Understanding whether ICI remains effective in older populations is critical but remains a challenge due to the underrepresentation of elderly patients in clinical trials [119]. Clinical outcomes of ICI in other under-represented populations have been comprehensively reviewed [120]. Recently, several studies have sought to specifically investigate the efficacy of ICI treatment in the aged population. Though most clinical studies have found that ICI shows similar efficacy in older and younger patients [79,121,122,123], some studies have reported conflicting findings suggesting that elderly patients may fare better or worse with ICI in specific contexts [82,124]. A recent meta-analysis [125] surveyed 30 randomized controlled trials to build a meta-cohort of 17,476 patients with various solid tissue malignancies. Analysis of survival showed that across cancer types, there was overall little to no difference in progression-free survival or overall survival in younger (<65 years) or older (≥65 years) patients [125]. The only statistically significant finding was slightly improved progression-free survival in younger patients with melanoma, but older melanoma patients still benefited significantly from ICI [125]. Importantly, although this study demonstrated no clinical difference between patients stratified into discrete age groups, others have shown that patients at the extremes of age demonstrate more variable response patterns [80]. Further evaluation of the impact of age on ICI efficacy is warranted, and consideration of treatment type, cancer type, and dosage may help delineate the impacts of aging and senescence on ICI efficacy.

### 5.2. CIinical Observations Regarding the Impact of Aging on Adverse Events

Conflicting evidence about the effects of age on immune-related toxicities resulting from irAEs suggests that there may be an impact of age on ICI toxicity, but that this effect may be modest. A retrospective study of 288 patients with multiple cancer types treated with ICI in Japan found that patients with more severe irAEs tended to be on average 2.7 years older, but that patients with irAEs tended to have improved overall response rates [80]. Other studies focused on specific types of cancer also vary in their findings about ICI efficacy in older patients: a study of 227 patients with head/neck squamous cell carcinoma found that patients aged 70 or older responded similarly to ICI compared to their younger counterparts and had a comparable toxicity profile [81]. Another study showed that anti-PD-1/anti-PD-L1 ICI treatment induced fewer overall toxicities in patients aged 70 or older, and that though older patients tended to experience more skin toxicities, younger patients tended to suffer from more endocrine toxicities secondary to ICI treatment [82]. In contrast, another study of anti-PD1 treatment in three different cancer types showed that patients aged 70 or more had slightly higher (though not statistically significant) rates of irAEs than patients younger than 65 [126]. Overall, though some studies found a statistically significant difference in the rate of irAEs in older patients, most studies concluded that these differences still warranted the use of these treatments in elderly patients, as the efficacy was comparable or better than in younger patients. These studies are summarized in Table 1.

## 6. Evidence of Interaction between Senescence and ICI Treatment

### 6.1. Senescence in Tumor Cells

Senescence has been demonstrated to prevent tumorigenesis and stimulate clearance of pre-malignant cells, suggesting a protective role by preventing cancer [45,127,128]. Loss of senescence upon malignant transformation is well documented [64]. However, induction of senescence in transformed malignant tumor cells has been associated with poor outcomes. Senescent tumor cells have been shown to promote invasion into the lymphovascular system and prevented apoptosis during migration through expression of senescence-associated E-cadherin [129,130]. On the other hand, senescent tumor cells may also stimulate an immunogenic response more than non-tumor cells. One study showed that C57/Bl6J mice aged between 8 and 16 weeks immunized with senescent B16 murine melanoma cells were more likely to successfully mount an immune response against tumorigenic B16 melanoma compared to immunization with non-senescent or even dying cells [131]. In the context of ICI treatment, another study found that PD-L1 expression in both mice and humans tends to be elevated more broadly in senescent cells [87]. This finding suggests that senescence in tumor cells may lead to improved outcomes from ICI treatment. On the other hand, another study in diffuse large B-cell lymphoma (DLBCL) showed that senescent DLBCL tumor cells could signal to macrophages that potentiate the senescence phenotype in other non-senescent tumor cells [44]. These senescent DLBCL tumor cells tended to express more PD-L1, which led to increased targeting of this population with anti-PD1 ICI treatment [44]. Thus, though senescence in pre-malignant cells may prevent malignant transformation, senescence in tumor cells may be linked to more aggressive phenotypes and stimulate invasion and migration, although immune response to senescent tumor cells in the context of ICI may be more potent. In summary, senescence can be pleiotropic, playing a critical role in preventing cancer in early life but potentially exacerbating cancer growth and treatment resistance in older individuals. The specific role of tumor cell senescence on ICI response should be further evaluated.

### 6.2. Senescence in the Immune Compartments

As previously discussed, senescence in peripheral immune cells has been demonstrated to be associated with age and diminished immune effector function. The accumulation of senescent immune cells in specific tissues may explain the onset of irAEs—indeed, one study demonstrated that T cells could be induced to senescence, characterized by loss of CD27 and CD28, after exposure to tumor cells [104,111]. Interestingly, senescent T cells driven by p16^INK4A^ expression also tend to express the exhaustion marker PD-1 [103,107], a key target of ICI. Senescent non-immune cells also tend to express more PD-L1 [87], and the production of SASP is sufficient to induce PD-L1 expression in non-senescent cells [87]. Complementary increases in the expression of PD-1 in aged T cells (mice aged 17.5 months) explain the observation by Wang et al. that the use of anti-PD-1 ICI treatment resulted in increased senescent cell clearance and overall cellular rejuvenation in a p16-TdTomato mouse model (C57/Bl6J) [88]. Wang et al. also found that treatment with anti-PD1 ICI treatment led to CD8+ T cell-mediated clearance of senescent cells and reversed age-related phenotypes including steatotic liver disease [88]. Though this observation does not directly indicate that senescence in the immune compartment was responsible for age-related phenotypes, it does suggest that targeting age-related exhaustion and senescence markers in the immune compartments, including the ICI target PD-1, may reverse age-associated phenotypes, which includes senescence.

Myeloid-derived suppressor cells (MDSCs) are another important immune population whose frequency drastically changes in the context of aging and senescence. In the context of aging and cancer, myelopoesis has been observed to increase with age in both rodents and humans [132,133], with a particular elevation in patients with a history of cancer [134]. Increased myeloid immune cell populations are linked to the inflammaging phenotype [54]. Patients treated with ICI therapy were shown to have a profound clinical response only with a reduction in the peripheral MDSC population, and an elevated MDSC count was predictive of failure to respond to ICI treatment [135,136]. MDSCs have been shown to respond strongly to interferon signaling, with one study showing that ensuring the type I interferon receptor IFNAR1 in MDSCs abrogated any suppressive activity resulting in strong anti-tumor immunity [137]. Another study showed that halting signaling via type I interferons resulted in enhanced suppressive activity of MDSCs and subsequent resistance to ICI treatment [138]. Though the exact impact of cellular senescence within MDSCs has yet to be explored, these cells are highly associated with the onset of age-related immune changes by modulating senescence phenotypes in surrounding cells. For example, one study found that MDSCs prevent senescence in cardiac myofibroblasts, resulting in age-related cardiac fibrosis [139]. Another study demonstrated that a subpopulation of MDSCs could induce senescence in CD8+ T cells through exosomal transfer of GPR84, a G-protein-coupled receptor that signals through p53 to halt cell proliferation [140]. Taken together, these findings suggest that age-related change in MDSC populations may affect response to ICI, either by direct impact on target cell types like CD8+ T cells, or indirectly through, for example, cytokine signaling.

### 6.3. Senescence in the Tumor Microenvironment

Stromal senescence may play a pro- or anti-tumor role in cancer development, progression, and treatment response. A breakthrough study by Krtolica et al. showed that co-culture with senescent fibroblasts could stimulate malignant transformation in non-malignant epithelial cells and hastened tumorigenesis in malignant cells [20]. A more recent study by Haston et al. found that in C57/Bl6J mice engineered to harbor a KRAS G12V mutation, lung tumor growth and proliferation was attenuated by age 10–12 months after clearance of senescent tumor-associated macrophages using the senolytic ABT-737 [141]. Clinically, Haston et al. also found senescent macrophages not only in the tumor microenvironments of human lung cancers but also in pre-cancerous lung lesions, suggesting that senescence does promote tumorigenesis in humans [141]. Cancer-associated fibroblasts in the tumor microenvironment have also been shown to promote invasion and epithelial disruption in oral squamous cell carcinoma [142], suggesting a key role of senescent cells in driving metastatic spread. Another in vitro study of numerous prostate and breast cancer cell lines suggested that extracellular vesicles produced by senescent cells promoted resistance to chemotherapeutic agents by upregulating expression of drug transport protein ABCB4 [143]. Taken together, these studies suggest that senescent cells in the tumor microenvironment play a pro-tumorigenic role and may enable tumors to migrate and metastasize, as well as to develop resistance to treatment.

In the context of ICI treatment, senescence in the tumor-adjacent stroma or in the tumor immune microenvironment may play a significant immunosuppressive role in response to ICI [131,144]. A recent study directly addressed the question of the impact of senescence on ICI response. Using a murine model that allowed for clearance of senescent cells from the tumor microenvironment, Maggiorani et al. showed that elimination of senescent cells diminished the immunosuppressive activity of myeloid cells and resulted in improved overall survival and response to ICI [145]. In this study, senescence in 12-week-old mice was induced with total body irradiation or by treatment with doxorubicin, and using the p16-3MR transgenic mouse model (C57/Bl6J), senescent cells were cleared upon treatment with ganciclovir, or using the senolytic drug ABT263 (navitoclax) [145]. These results suggest a potential role for senolytic treatments in concert with ICI to improve outcomes. Clinical trials evaluating the safety and efficacy of senolytic drugs are currently underway, although to date, no trials have explored combining senolytic treatments with ICI therapy, partly because of the unpredictable balance between pro- and anti-tumoral effects of senescence. Clinical trials evaluating the potential interaction between aging and ICI treatment are summarized in Section 5.

### 6.4. The Impact of Senescence on Immune-Related Adverse Events

Senescence may also be linked to the incidence, location, timing, and duration of immune-related adverse events (irAEs) secondary to ICI treatment. Conflicting evidence in clinical observations has indicated that elderly patients may be more susceptible to adverse events [81,126], especially in specific tissues such as the skin. Senescent cells secrete pro-inflammatory cytokines as part of the SASP, including IL-1, IL-6, IL-8, interferon gamma, and others [33,34]. Recent clinical studies have shown that blocking IL-6 prevents and ameliorates the onset of irAEs [43,146]. On the other hand, another study found that low serum IL-6 was prognostic of more frequent and severe irAEs [147]. Another attribute that may explain age-associated irAE incidence is the increased expression levels of PD-L1 in senescent tumor microenvironmental cells or in non-tumor stromal cells in patients with high senescent cell burden [87]. These cells may be cleared with ICI treatment, and indeed, one study showed that treatment with ICI is linked to the clearance of senescent cells [88]. Immune-mediated clearance of senescent tissue by the immune system has been shown to be relatively innocuous in transgenic and senolytic-treated mice [66,70,148], but the impact of profound senescence clearance in aged human patients has not been fully explored.

The clearest study linking aging and senescence to the onset of immune-related adverse events is that by Tsukamoto et al. [149]. In this comprehensive study of the impact of aging on immune checkpoint response and toxicity, the authors develop a murine model for studying the interaction between age and irAEs secondary to anti-PD1. Old (>18 months) and young (<3 months) mice (C57/Bl6-J and Balb-c) were orthotopically injected with melanoma or colon cancer cell lines and subsequently treated with anti-PD1. In this model, older mice appeared to have diminished response to anti-PD1 treatment but tended to have CD4+ T cell-mediated elevated immune infiltration in non-tumor tissues, including the lung, liver, and kidneys, consistent with the histological hallmarks of irAEs. The authors also emphasized the role of IL-21 and CXCL13 in the onset of irAEs. An important limitation of this study is the sole use of anti-PD1 ICI treatment. However, the authors mention in the supplement and discussion that though the addition of anti-CTLA4 to anti-PD1 in their murine model exacerbated the irAE incidence, the underlying biology of CD4+ cell-mediated IL21 production and CXCL13 elevation are not seen in single-agent anti-CTLA4 treatment. This finding may suggest that the mechanism for irAEs on anti-CTLA4 may be distinct and may synergize with anti-PD1 irAEs.

## 7. Conclusions and Future Directions

Cellular senescence is pleiotropic in the context of cancer and can play multiple roles in the development, progression, and spread of cancer. Although the exact roles of senescence on immunotherapy have not been elucidated, evidence suggests that senescence in specific compartments can influence the efficacy and toxicity of immune checkpoint inhibitor treatment. Senescence in tumor cells can result in slowed growth but can also promote invasion and metastasis and confer treatment resistance to anti-cancer therapies, which may include immunotherapy. In stromal cells within the tumor microenvironment, senescence can lead to the production of pro- and anti-inflammatory cytokines, which has been shown to promote resistance to immunotherapy [145]. Senescence in non-tumor tissue may predispose those tissues to the onset of immune-related adverse events [14]. Finally, senescence in immune cells, which express several exhaustion markers including PD-1 [103], may potentiate the effects of immune checkpoint inhibitor treatment, while exacerbating immune-related adverse events [150].

To our knowledge, several pre-clinical studies have directly addressed the impact of aging and senescence on immune checkpoint inhibitor efficacy and toxicity. Maggiorani et al. [145] showed that senescence elimination could overcome resistance to ICI treatment. Contrastingly, Hao et al. [76] showed that amplifying the SASP could overcome ICI resistance in ovarian cancer. Tsukamoto et al. [149] demonstrated a potential mechanism involving CD4+ T cells and the cytokines IL-21 and CXCL13, which are notably components of the SASP, through which older individuals would have a higher incidence of irAEs. Further studies are required to evaluate the mechanisms by which senescence can impact immunotherapy treatment.

To investigate the impact of senescence on ICI efficacy and toxicity, murine models of senescence and aging could be employed. Several model organisms used to study p16^INK4A^-driven senescence include the P16-3MR and INK/ATTAC models [58,66], which enable the clearance of senescent cells induced by p16^INK4A^. Conditional depletion of cells mediated by alternative forms of senescence, such as p21^CIP^- or p14^ARF^-mediated senescence, can also provide further insights into the effects of different pathways in modulating senescence and effects of ICI treatment. Tumor cells of various cancer types could be introduced in these animal models to better understand the context-specific nature of adverse events in the elderly population. Future studies may explore the specific clearance of senescent stromal or tumor cells in a murine model and the impact of this clearance on ICI efficacy and toxicity, while controlling for environmental or genetic factors.

In addition to the use of transgenic animal models to study the mechanistic effects of senescence clearance, therapeutic options for senescence clearance are also feasible and would lead to more translatable findings. Senolytic treatments, including navitoclax, dasatinib, quercetin, fisetin, and others, have been demonstrated to eliminate senescent cells in rodents and humans [71,72,73,74,75]. Development of the next generation of senolytic and senomodulatory treatments has also begun and holds promise in more specifically and potently targeting senescent cells. These treatments and future directions of senolytic and senomodulatory therapy have been reviewed elsewhere [151]. As of June 2024, no active clinical trials are investigating the impact of senescence clearance or modulation on immunotherapy efficacy or toxicity. A randomized, controlled clinical trial in which patients would be assigned either ICI alone or ICI in combination with a senolytic or senomorphic agent would determine whether clearance of senescent cells would be beneficial or detrimental for patients receiving immunotherapy such as ICI and whether senolytic treatments could be safe and effective in combination with ICI treatment.

In summary, aging and cellular senescence may influence immune checkpoint inhibitor response and toxicity within the tumor, the tumor microenvironment, or the immune system. Though clinical evidence regarding the relationship between aging and immune checkpoint inhibitor treatment outcomes is conflicting, preclinical studies have suggested an important role for aging and cellular senescence in modulating the efficacy of immune checkpoint inhibitor therapy and the incidence of immune-related adverse events. Further mechanistic studies and clinical trials evaluating the effects of senescent cell clearance are required to evaluate the full scope of interaction between immune checkpoint inhibitor treatment and cellular senescence and to establish a refined approach to combinatory treatments.

## Figures and Tables

**Table 1:** Summary of key clinical studies on the impact of aging and senescence on ICI efficacy and toxicity.

Study	Disease Context	Patient Demographics	Therapeutics Investigated	Findings
Marrone et al., 2018 [79]	Non-small cell lung cancer	Total N = 275 Age: <75 y.o. (92.7%), ≥75 y.o. (7.2%) Sex: N/D * Race: N/D	Anti-PD-1 Anti-PD-L1	Efficacy: N/S * Toxicity: N/D
Baldini et al., 2020 [82]	Melanoma, non-small-cell lung cancer, renal cell carcinoma, head/neck squamous cell carcinoma, Merkel cell carcinoma, others	Total N = 603 Age: <70 y.o. (68.3%), ≥70 y.o. (31.7%) Sex: Female (44%) Race: N/D	Anti-PD-1 Anti-PD-L1	Efficacy: PFS slightly higher in ≥70 y.o. (but N/S) Toxicity: Skin and multiple irAEs significantly more likely in ≥70 y.o.
Truong et al., 2018 [121]	Non-small-cell lung cancer, melanoma, others	Total N = 776 Age: 65–79 y.o. (83%), ≥80 y.o. (17%) Sex: Female (42%) Race: Non-caucasian (29%)	Anti-PD-1 Anti-PD-L1	Efficacy: N/S Toxicity: N/S
Saleh et al., 2021 [81]	Head/neck squamous cell carcinoma	Total N = 226 Age: <70 y.o (70.4%), ≥70 y.o. (29.6%) Sex: Female (18%) Race: N/D	Anti-PD-1 Anti-PD-L1 Anti-CTLA4 Anti-KIR ** Vaccination	Efficacy: PFS and ORR significantly higher in ≥70 y.o. Toxicity: All adverse events of any grade significantly more likely in ≥70 y.o., but N/S in grade 3+ irAEs
Singh et al., 2016 [126]	Renal cell carcinoma, melanoma, non-small-cell lung cancer	Total N = 1030 Age: <65 y.o. (59.8%), 65–70 y.o. (19.6%), ≥70 y.o. (20.6%) Sex: N/D Race: N/D	Anti-PD-1	Efficacy: N/D Toxicity: All grade and grade 3–5 adverse events occurred more frequently in ≥65 y.o., but statistical significance N/D
Schonfeld et al., 2022 [122]	Melanoma	Total N = 4489 Age: 66–84 y.o. (100%) Sex: Female (33.1%) Race: Non-Caucasian (0%)	Anti-PD-1 Anti-PD-L1 Anti-CTLA-4 Combinations	Efficacy: N/D Toxicity: Incidence of irAEs in 66–84 y.o. is higher than without ICI
Matsuoka et al., 2020 [80]	Gastric, lung, renal cell carcinoma, head and neck squamous cell carcinoma, melanoma, Hodgkin’s lymphoma, others	Total N = 280 Age: 22–87 y.o. (100%)	Anti-PD-1	Efficacy: N/D Toxicity: Patients with irAEs were significantly older than patients without irAEs

* N/D = not discussed; N/S = no statistically significant difference. ** KIR = Killer immunoglobulin-like receptors.
